# Leukotriene B_4_ receptors mediate the production of IL‐17, thus contributing to neutrophil‐dominant asthmatic airway inflammation

**DOI:** 10.1111/all.13789

**Published:** 2019-04-04

**Authors:** MyungJa Ro, Sun‐Young Kwon, Jae‐Hong Kim

**Affiliations:** ^1^ Department of Biotechnology, College of Life Sciences and Biotechnology Korea University Seoul South Korea

To the editor,

Asthma phenotypes can be classified according to the inflammatory immune cell type that infiltrates the airways, such as eosinophilic, neutrophilic, or mixed eosinophilic/neutrophilic inflammation.^1^ Generally, eosinophilic inflammation mostly occurs in mild‐to‐moderate asthma, while neutrophilic inflammation is seen in more severe asthma phenotypes.^1^ Steroids, the cornerstone medication for asthma, were reported to increase neutrophil‐dominant airway inflammation by interrupting neutrophil apoptosis and enhancing neutrophil activation.^1^ Thus, in some asthmatic patients, an increase in neutrophil number in the sputum is associated with persistent asthma and acute asthma exacerbations^2^; this condition is called steroid‐resistant severe asthma. Recently, IL‐17 was shown to be associated with severe neutrophilic asthma development and exacerbation, and IL‐17 levels in serum and sputum were shown to increase with disease severity.^3^, ^4^ Meanwhile, a previous study demonstrated that leukotriene B4 (LTB_4_) is present at higher concentrations in sputum of patients with severe asthma, who have relatively pronounced neutrophil influx, than in those with mild asthma.^5^ Furthermore, LTB_4_ receptors, BLT1 and BLT2, were suggested to contribute to the development of asthmatic airway inflammation.^6^, ^7^ In the present study, we examined whether BLT1/2‐cascade plays a role in the development of neutrophil‐dominant airway inflammation.

Details for materials and methods are provided in this article's online supporting information (Data S1).

To generate a neutrophil‐dominant pulmonary inflammation model, mice were immunized with 1 or 10 μg of LPS and 75 μg of OVA on days 0, 1, 2, and 7 and then challenged with 50 μg of OVA on days 14, 15, 21, and 22. Mice were killed on day 24 (Figure S1).^8^ Lung inflammation increased upon immunization with LPS/OVA compared to controls, as observed in the histology analysis (Figure S1). Then, we tested the efficacy of steroid (dexamethasone) in this model. Airway inflammation, mucus secretion, and the recruitment of total immune cells, neutrophils, eosinophils, and lymphocytes were significantly suppressed by dexamethasone in OVA‐induced mice (Figure S1) but were only partially reduced by dexamethasone in LPS/OVA‐induced mice (Figure S1). Thus, these results suggested that our LPS/OVA‐induced airway inflammation model is suitable for studying neutrophil‐dominant allergic pulmonary inflammation that is less sensitive to dexamethasone (Figure S2). Previously, IL‐17 was shown to be closely associated with the pathogenesis of severe neutrophilic asthmatic inflammation, and IL‐17 levels in serum and sputum increase with disease severity.^3^ In agreement with these previous reports, IL‐17 levels were significantly increased in BALF from mice with LPS/OVA‐induced pulmonary inflammation (Figure S3). The levels of IL‐4, IL‐5, and IL‐13 were not increased in BALF of LPS/OVA‐induced mice (Figure S4). To examine the contribution of IL‐17, an anti‐IL‐17 neutralizing antibody (100 μg/mouse) was intraperitoneally administered 1 hour before each challenge. Airway inflammation, mucus secretion, and the infiltration of immune cells were markedly reduced by the anti‐IL‐17 neutralizing antibody (Figures S2, S3). Together, these results suggested that IL‐17 contributes to neutrophil‐dominant airway inflammation and inflammatory immune cell recruitment.

LTB_4_ levels are higher in exhaled breath condensates of severe asthma patients than in those of mild asthma patients.^5^ Therefore, we examined the role of the LTB_4_ receptors BLT1 and BLT2 in IL‐17 production in the neutrophil‐dominant pulmonary inflammation model. We observed increased expression of BLT1 and BLT2 in the lungs of LPS/OVA‐induced mice (Figure 1A). To this aim, the BLT1 inhibitor U75302 (10 μg/mouse) or the BLT2 inhibitor LY255283 (10 mg/kg) was intraperitoneally injected 1 hour before every challenge. Airway inflammation was markedly reduced by U75302 or LY255283, as determined by histological analysis and quantitative analysis of inflammation scores (Figure 1B). Additionally, mucus secretion and the accumulation of total immune cells, neutrophils, eosinophils, and lymphocytes in BALF were highly enhanced by LPS/OVA administration, and this effect was suppressed by U75302 or LY255283 (Figure 1B,C). In addition, the increased IL‐17 level was suppressed by treatment with the BLT1 or BLT2 inhibitor (Figure 1D). Together, these results suggested that BLT1 and BLT2 contribute to IL‐17 production and airway inflammation in neutrophil‐dominant pulmonary inflammation. LTB_4_ and 12(*S*)‐hydroxyeicosatetraenoic acid (12(*S*)‐HETE) are synthesized by the catalytic action of 5‐lipoxygenase (5‐LO) and 12‐lipoxygenase (12‐LO), respectively, and these lipid metabolites were shown to interact with BLT1/2.^9^ Next, we examined whether an autocrine or paracrine LTB_4_/12(*S*)‐HETE‐BLT1/2 cascade is implicated in IL‐17 production in response to LPS/OVA‐induced airway inflammation. To inhibit 5/12‐LO, mice received an oral dose of the 5‐LO inhibitor MK886 (5 mg/kg) or the 12‐LO inhibitor baicalein (75 mg/kg) 1 hour before every challenge. We observed that 5‐/12‐LO expression in the lungs and LTB_4_ and 12(*S*)‐HETE serum levels were elevated in LPS/OVA‐induced pulmonary inflammation group (Figure 2A,B). Histology analysis showed that MK886 or baicalein reduced airway inflammation compared to control (Figure 2C). Additionally, the administration of MK886 or baicalein significantly reduced the infiltration of total immune cells, neutrophils, eosinophils, lymphocytes, macrophages, and the levels of IL‐17 as well as mucus secretion (Figure 2C,D,E). Together, these results suggested that the LPS/OVA‐induced production of IL‐17 and stimulation of airway inflammation are dependent on the 5‐/12‐LO‐BLT1/2 cascade in the neutrophil‐dominant pulmonary inflammation model. Then, we examined whether the 5‐/12‐LO‐BLT1/2 cascade is associated with NF‐κB activation‐mediated IL‐17 production in our model. We observed that MK886, baicalein, U75302, or LY255283 suppressed the NF‐κB activation in the lungs of LPS/OVA‐treated mice (Figure S5). Also, NF‐κB inhibitor bay11‐7082 suppressed airway inflammation and IL‐17 production in the LPS/OVA‐treated mice (Figures S2, S5). These results suggested that NF‐κB activation lies downstream of 5‐/12‐LO‐BLT1/2 in the neutrophil‐dominant pulmonary inflammation model.

**Figure 1:**
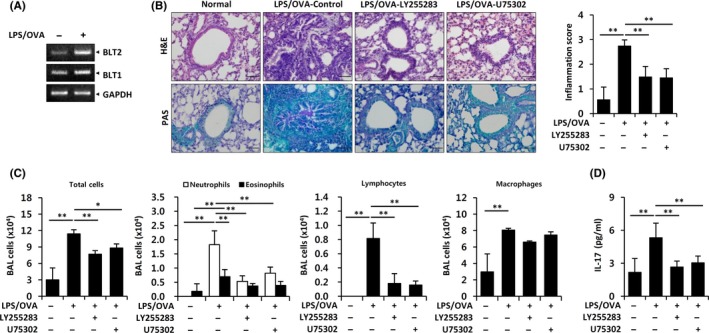
The LTB_4_ receptors BLT1 and BLT2 are critical for neutrophil‐dominant airway inflammation and IL‐17 production. A, RNA was isolated from lungs, and BLT1 and BLT2 transcript levels were assessed by RT‐PCR. B, The fixed lungs were stained with H&E and PAS. Peribronchial and perivascular lung inflammation was measured and scored. C, Immune cells in BALF were obtained using cytospin and stained with H&E. D, The levels of IL‐17 in BALF were analyzed using ELISA. All quantitative data are expressed as the mean ± SD. *P < 0.05, **P < 0.01

**Figure 2:**
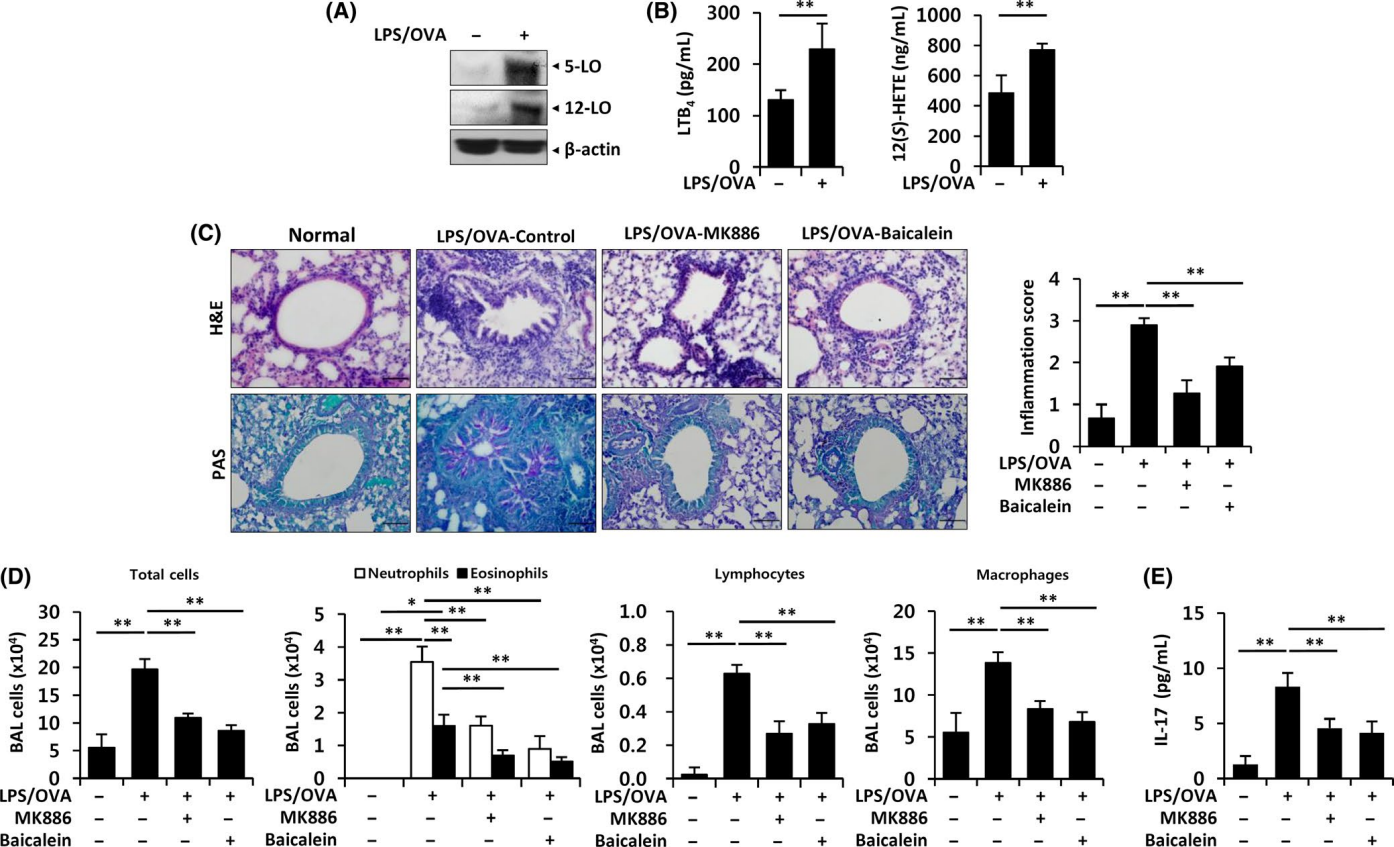
Upregulation of BLT2 ligands, LTB_4 _and 12(*S*)‐HETE, is associated with neutrophil‐dominant airway inflammation and IL‐17 production. A, Protein was isolated from lungs, and 5‐/12‐lipoxygenase was assessed by Western blotting. B, LTB_4_ and 12(*S*)‐HETE levels in serum were analyzed using ELISAs. C, The fixed lungs were stained with H&E and PAS. Peribronchial and perivascular lung inflammation was measured and scored. D, Immune cells in BALF were obtained using cytospin and stained with H&E. E, The levels of IL‐17 in BALF were analyzed using ELISA. All quantitative data are expressed as the mean ± SD. *P < 0.05, **P < 0.01 [Color figure can be viewed at http://wileyonlinelibrary.com]

In summary, we found that BLT1/2 play critical roles in the development of neutrophil‐dominant pulmonary inflammation and identified IL‐17 as a key cytokine synthesized through the BLT1/2‐cascade. We found that 5‐/12‐LO lie upstream of BLT1/2, and NF‐κB is downstream of the 5‐/12‐LO‐BLT1/2 cascade and mediates IL‐17 production in neutrophil‐dominant airway inflammation. Taken together, our findings demonstrate that 5‐/12‐LO‐BLT1/2‐NF‐κB‐IL‐17 signaling potentially exacerbates neutrophil‐dominant airway inflammation. This report is the first on the role of LTB_4_ receptors in a neutrophil‐dominant pulmonary inflammation model, and our results may provide a new perspective for the development of therapeutics for patients with asthma who are resistant to steroid treatment.

## CONFLICTS OF INTEREST

The authors declare that they have no conflicts of interest.
