# Supplementary material for: Leukotriene B4 receptors mediate the production of IL‐17, thus contributing to neutrophil‐dominant asthmatic airway inflammation
Source: Allergy. 2019 Apr 4;74(9):1797–9. doi: 10.1111/all.13789 (PMC6790678; doi:10.1111/all.13789)
Supplement: Supplementary file 1 [file ALL-74-1797-s001.docx]

1. **Figure S1. Dampened response to steroids in neutrophil-dominant airway inflammation.**


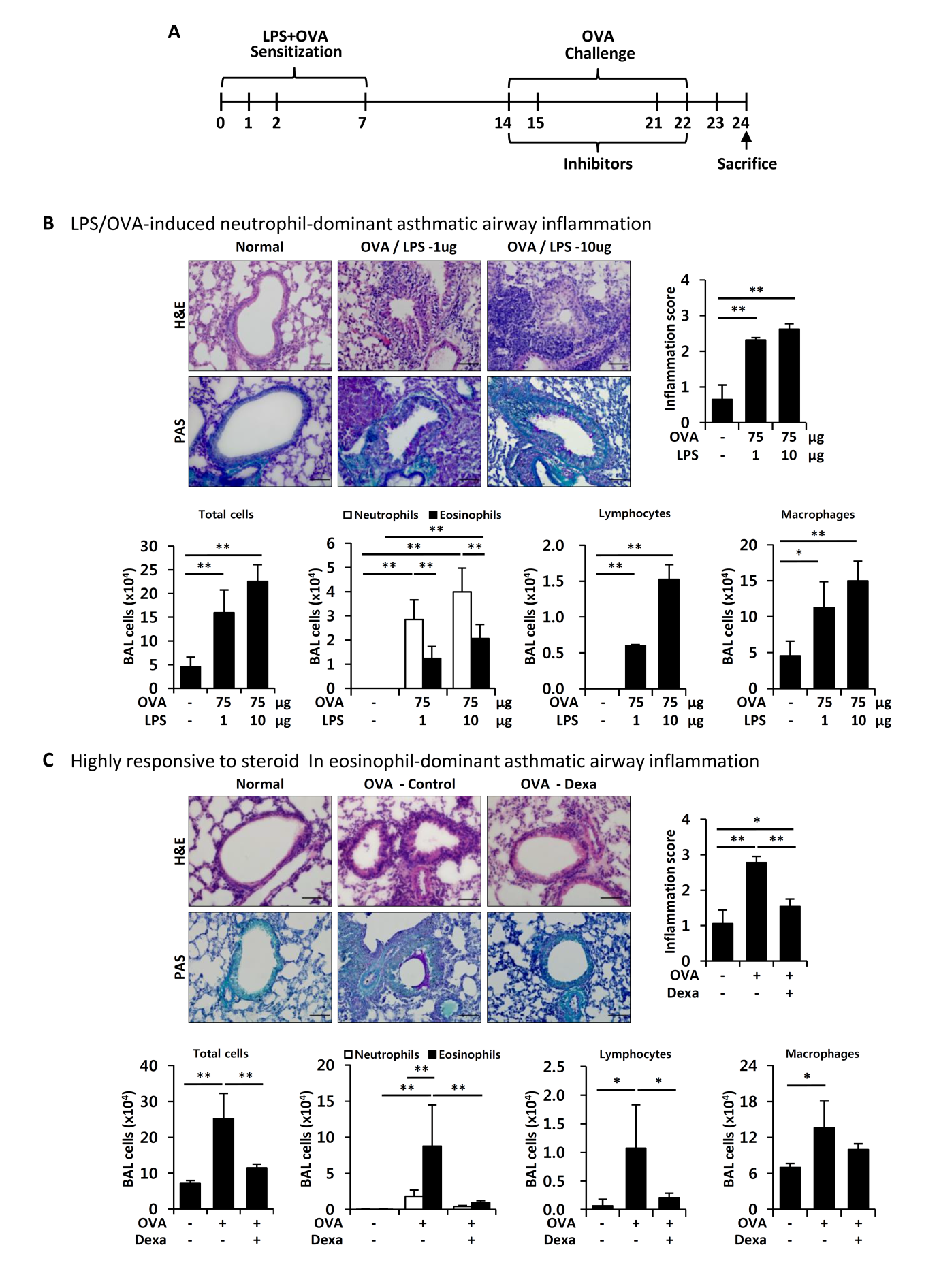


1.
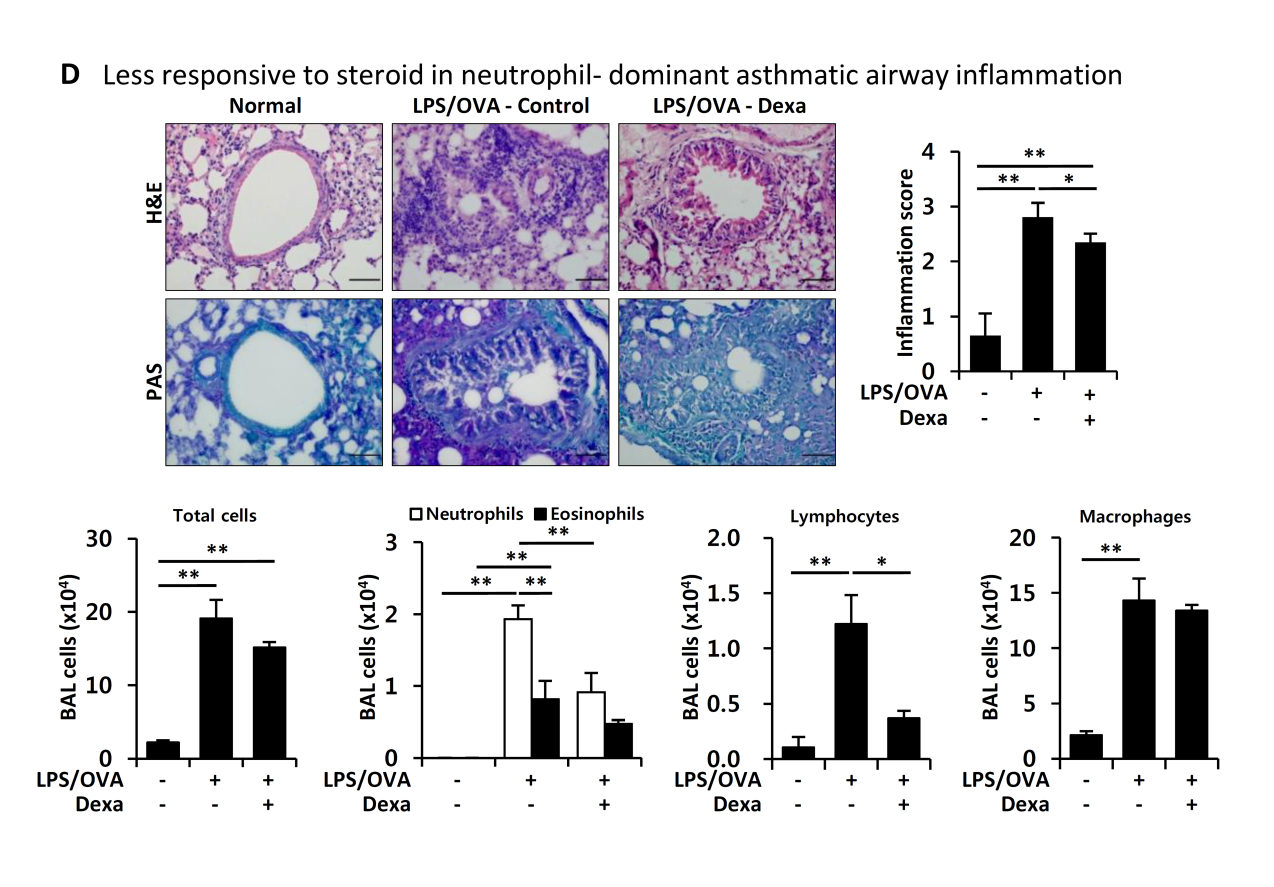

2. (A) Schedule of neutrophil-dominant airway inflammation model generation. (B and D) Airway inflammation was induced by immunization with OVA (75 μg) and LPS (1 or 10 μg) and challenge with OVA (50 μg) (n=3–5 per group). (C) Eosinophil-dominant airway inflammation was induced by immunization with 5 µg of OVA and 1 mg of alum and then challenge with 5 µg of OVA and 0.1 µg LPS (n=3–5 per group). (C and D) Dexamethasone (1 mg/kg) and vehicle were administered orally 1 h before every challenge. The lungs were excised, fixed and stained with H&E and PAS. Peribronchial and perivascular lung inflammation was measured and scored. Total immune cells, neutrophils, eosinophils, lymphocytes and macrophages in BALF were obtained using cytospin and stained with H&E. All quantitative data are expressed as the mean ± SD. **P*<0.05, ***P*<0.01.
