# Supplementary material for: Leukotriene B4 receptors mediate the production of IL‐17, thus contributing to neutrophil‐dominant asthmatic airway inflammation
Source: Allergy. 2019 Apr 4;74(9):1797–9. doi: 10.1111/all.13789 (PMC6790678; doi:10.1111/all.13789)
Supplement: Supplementary file 2 [file ALL-74-1797-s002.docx]

**Figure S2. Percent reduction of inflammation score and immune cell recruitment in response to inhibitors in OVA- or LPS/OVA-induced airway inflammation model.**

**
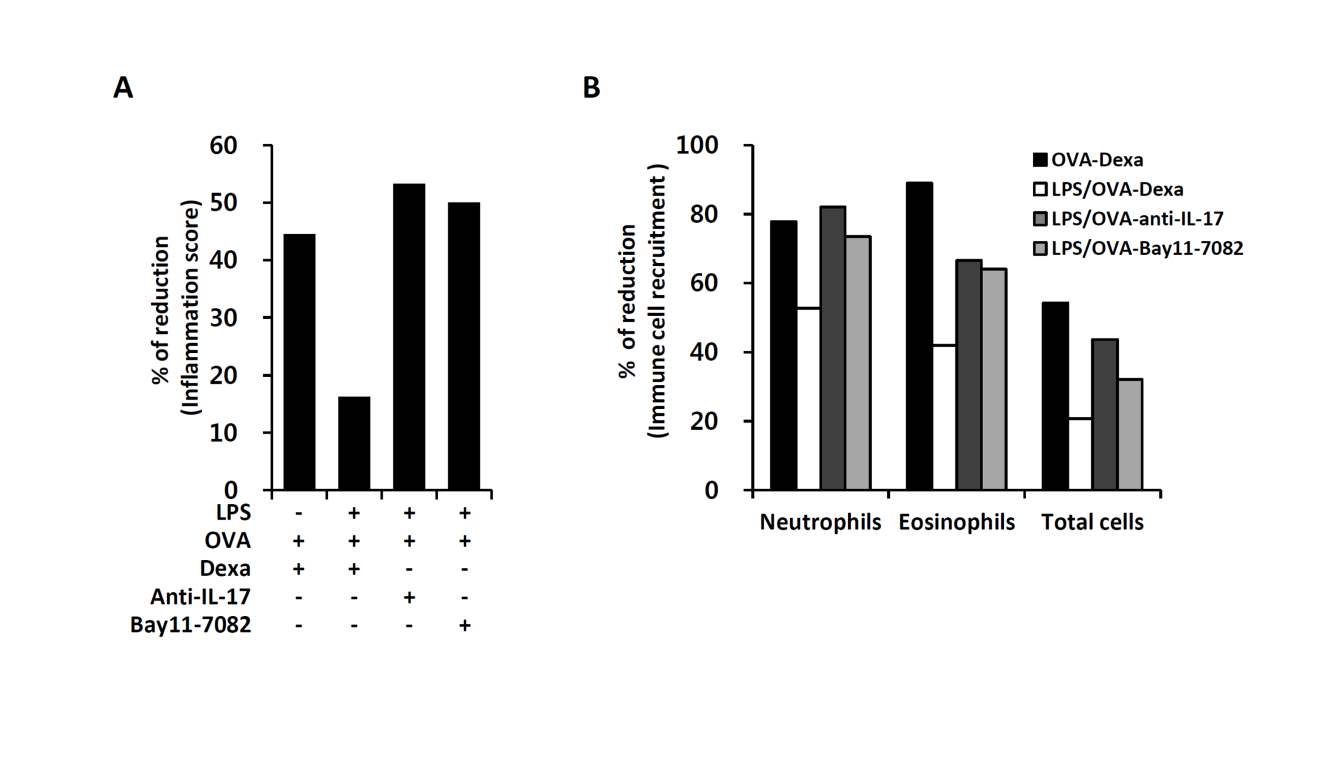
**

Dexamethasone (Dexa) treatment reduced airway inflammation relatively less in LPS/OVA-induced model than in OVA-induced model. In LPS/OVA-induced model, the percent (%) of reduction in inflammation was more evident by anti-IL-17 or Bay11-7082 than by DEX treatment. The % of reduction was calculated by finding the difference between value of OVA or LPS/OVA-control group and value of OVA or LPS/OVA-inhibitor group (value_control_-value_inhibitor_), then the result was divided by value of OVA or LPS/OVA-control group (value_control_). Lastly, the result was multiplied by 100. The formula was expressed as (value_control_-value_inhibitor_)/ value_control_ x 100.
