# Supplementary material for: Leukotriene B4 receptors mediate the production of IL‐17, thus contributing to neutrophil‐dominant asthmatic airway inflammation
Source: Allergy. 2019 Apr 4;74(9):1797–9. doi: 10.1111/all.13789 (PMC6790678; doi:10.1111/all.13789)
Supplement: Supplementary file 3 [file ALL-74-1797-s003.docx]

**Figure S3. IL-17 is critical for neutrophil-dominant airway inflammation.**


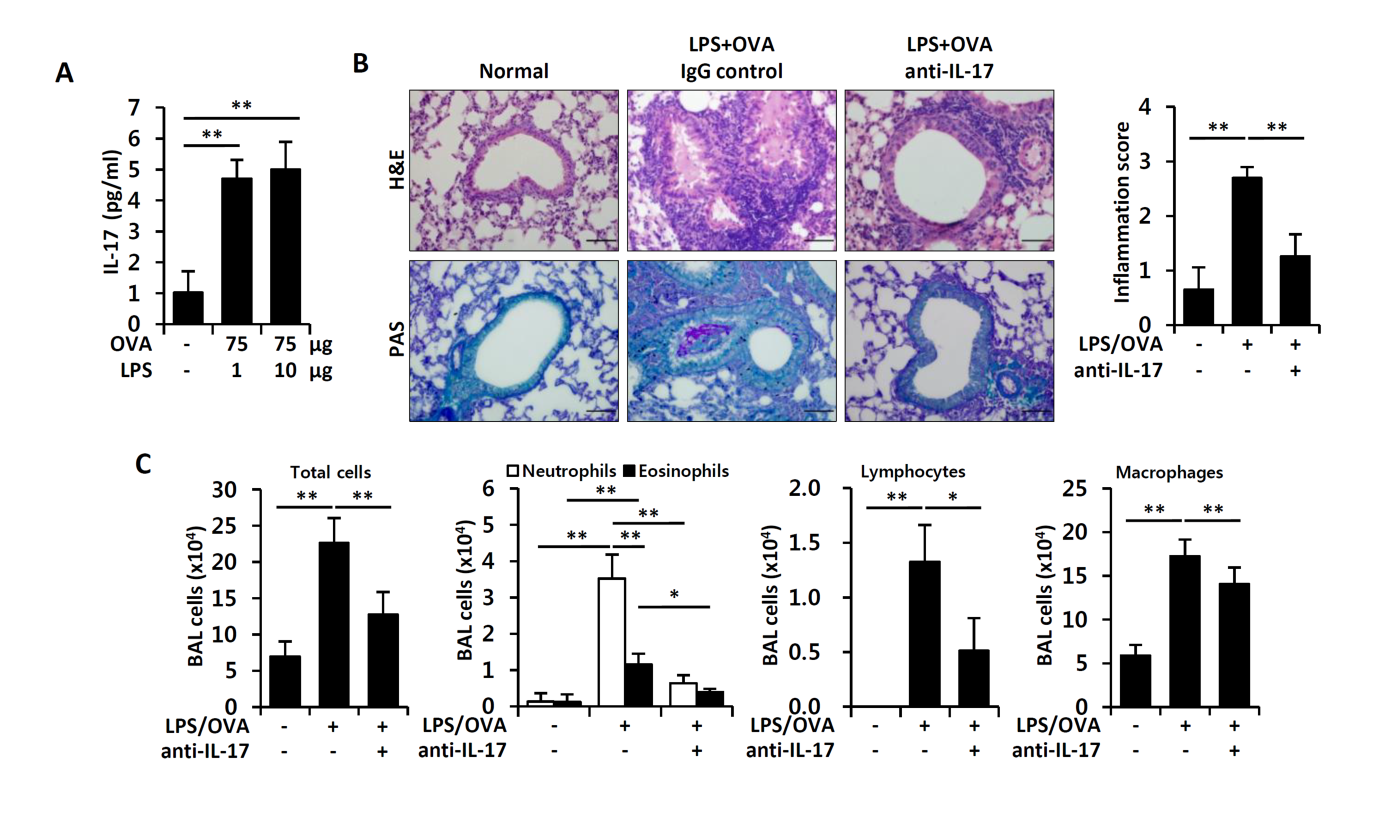


Airway inflammation was induced by immunization with 1 μg of LPS/75 μg of OVA and subsequent challenge with 50 μg of OVA. For the IL-17 inhibition experiment, mice received an i.p. injection of 100 μg of control IgG1 or anti-IL-17A antibody 1 h before every challenge (n=3–5 per group). (A) The levels of IL-17 in BALF were analyzed using ELISA. (B) The lungs were excised, fixed and stained with H&E and PAS. Peribronchial and perivascular lung inflammation was measured and scored. (C) Total immune cells, neutrophils, eosinophils, lymphocytes and macrophages in BALF were obtained using cytospin and stained with H&E. All quantitative data are expressed as the mean ± SD. **P*<0.05, ***P*<0.01.
