# Supplementary material for: Leukotriene B4 receptors mediate the production of IL‐17, thus contributing to neutrophil‐dominant asthmatic airway inflammation
Source: Allergy. 2019 Apr 4;74(9):1797–9. doi: 10.1111/all.13789 (PMC6790678; doi:10.1111/all.13789)
Supplement: Supplementary file 4 [file ALL-74-1797-s004.docx]

**Figure S4.The levels of Th2 cytokines were not increased in the BALF of LPS/OVA-induced mice**


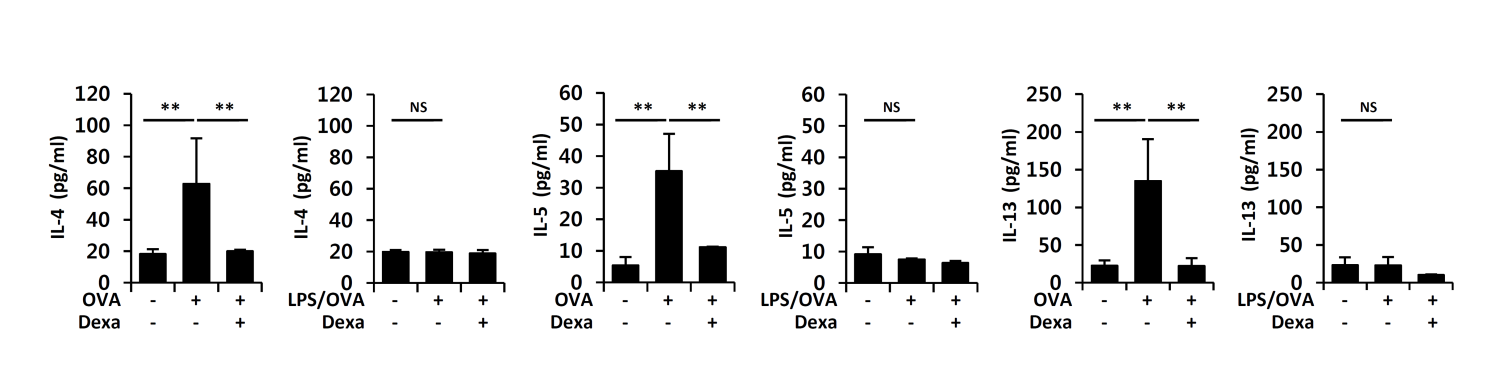


For neutrophil-dominant airway inflammation model, airway inflammation was induced by immunization with OVA (75 μg) and LPS (1 μg) and challenge with OVA (50 μg) (n=3–5 per group). And, the eosinophil-dominant airway inflammation was induced by immunization with 5 µg of OVA and 1 mg of alum and then challenge with 5 µg of OVA and 0.1 µg LPS (n=3–5 per group). Dexamethasone (1 mg/kg) and vehicle were administered orally 1 h before every challenge. The levels of IL-4, IL-5 and IL-13 in BALF were analyzed using ELISA. All quantitative data are expressed as the mean ± SD. **P*<0.05, ***P*<0.01.
