# Supplementary material for: Leukotriene B4 receptors mediate the production of IL‐17, thus contributing to neutrophil‐dominant asthmatic airway inflammation
Source: Allergy. 2019 Apr 4;74(9):1797–9. doi: 10.1111/all.13789 (PMC6790678; doi:10.1111/all.13789)
Supplement: Supplementary file 5 [file ALL-74-1797-s005.docx]

1. **Figure S5. NF-κB lies downstream of the 5-/12-lipoxygenase-BLT1/2 axis and mediates IL-17 production in neutrophil-dominant airway inflammation.**


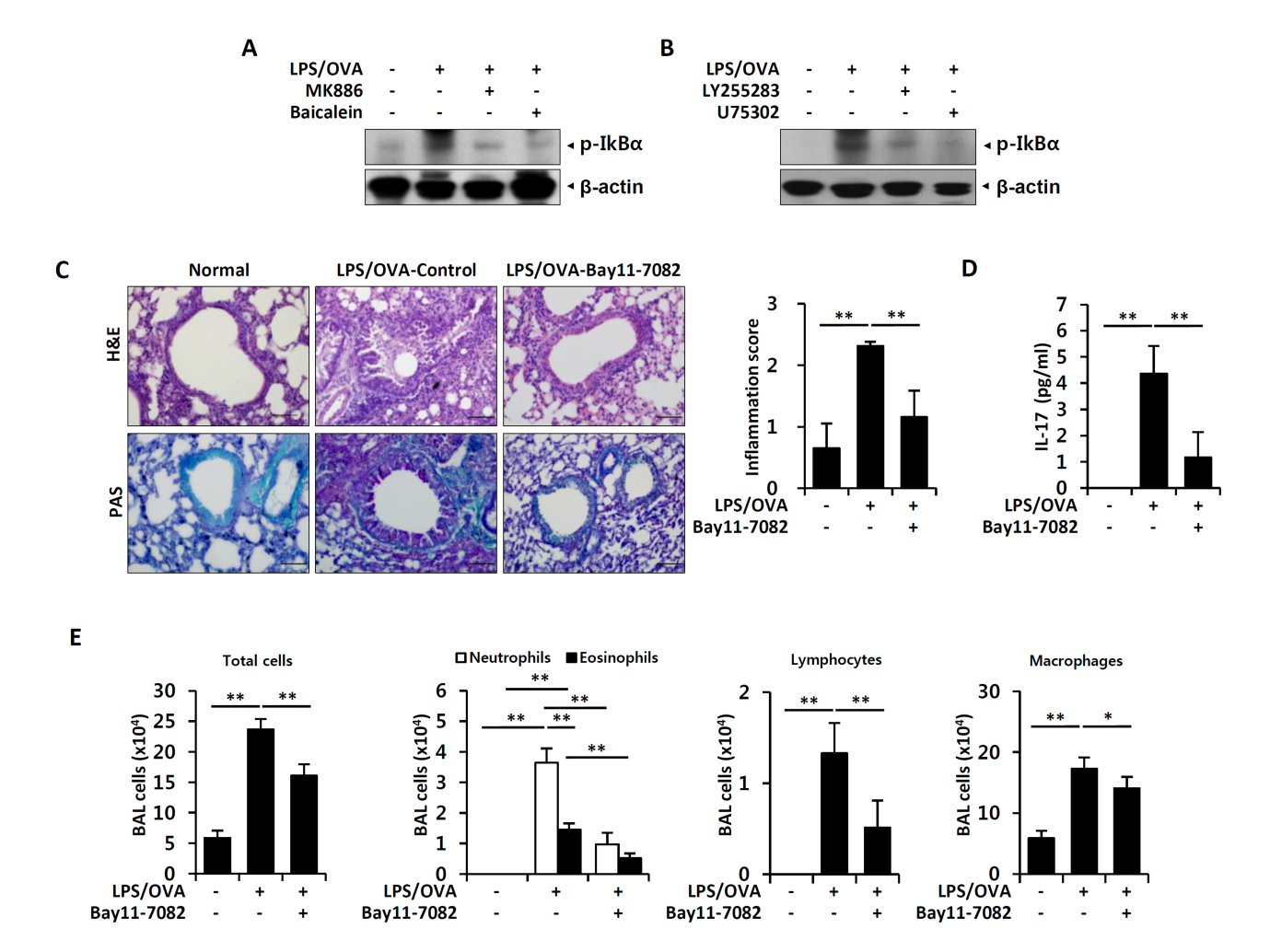


Airway inflammation was induced by immunization and challenge as described in Figure S1. MK886 (5 mg/kg), baicalein (75 mg/kg) or control (0.1 % methylcellulose) was administered orally 1 h before every challenge. LY255283 (10 mg/kg), U75302 (10 μg/mouse), Bay11-7082 (5 mg/kg) or control (DMSO) was administered via i.p. injection 1 h before every challenge (n=3–5 per group). (A and B) The mouse lungs were homogenized, protein was isolated, and p-IκBα levels were assessed by western blotting. (C) The lungs were excised, fixed and stained with H&E and PAS. Peribronchial and perivascular lung inflammation was measured and scored. (D) Levels of IL-17 in BALF were analyzed using ELISA. (E) Total immune cells, neutrophils, eosinophils, lymphocytes and macrophages in BALF were obtained using cytospin and stained with H&E. All quantitative data are expressed as the mean ± SD. **P*<0.05, ***P*<0.01.
