# Supplementary material for: Leukotriene B4 receptors mediate the production of IL‐17, thus contributing to neutrophil‐dominant asthmatic airway inflammation
Source: Allergy. 2019 Apr 4;74(9):1797–9. doi: 10.1111/all.13789 (PMC6790678; doi:10.1111/all.13789)
Supplement: Supplementary file 6 [file ALL-74-1797-s006.docx]

**Supporting information**

**Materials and methods**

***Reagents***

Lipopolysaccharide (LPS, Escherichia coli serotype O55:B5), ovalbumin (OVA), dimethyl sulfoxide (DMSO), dexamethasone and methylcellulose were obtained from Sigma-Aldrich (St. Louis, MO, USA). MK886 and Bay11-7082 were purchased from Calbiochem (La Jolla, CA, USA). Baicalein was purchased from Enzo Life Sciences (Farmingdale, NY, USA). U75302 and LY255283 were obtained from Cayman Chemical (Ann Arbor, MI, USA). IgG isotype control and anti-IL-17 neutralizing antibodies (clone 50104) were obtained from R&D Systems (Minneapolis, MN, USA).

1. ***LPS/OVA-induced neutrophil-dominant airway inflammation***

Female BALB/c mice (8-10 wk old; 18–20 g) were obtained from Orient Bio (Seoungnam, Korea). Sensitization and challenge were performed as previously described ([1](#_ENREF_1)). The protocol used in this study is shown in Figure 1A. Briefly, mice were intranasally sensitized with 75 μg of OVA and different doses of LPS on days 0, 1, 2, and 7 and then challenged intranasally with 50 μg of OVA on days 14, 15, 21, and 22. The mice were sacrificed on day 24 to assess airway inflammation ([1](#_ENREF_1)). For the inhibition experiments, LY255283 (10 mg/kg), U75302 (10 μg/mouse), Bay11-7082 (5 mg/kg) or vehicle control (DMSO) was administered via intraperitoneal (i.p.) injection 1 h before every challenge. In addition, some mice received an oral dose of MK886 (5 mg/kg), baicalein (75 mg/kg), dexamethasone (1 mg/kg) or vehicle control (0.1 % methylcellulose) 1 h before challenge. For the IL-17 inhibition experiment, mice were treated with either 100 μg of control IgG1 or anti-IL-17A antibody via i.p. injection 1 h before every challenge. Animals were housed under 12:12 h light/dark conditions at a density of four to five mice per static polycarbonate microisolator cage containing disposable bedding. Wire-lidded food hoppers within the cages were filled to capacity with rodent chow, and the mice had access to bottle-supplied water. The study was carried out in strict accordance with the recommendations of the Guide for the Care and Use of Laboratory Animals of Korea University. The protocol was approved by the Committee on the Ethics of Animal Experiments of Korea University.

1. ***OVA-induced mild eosinophil-dominant airway inflammation***

BALB/c mice were sensitized with an i.p. injection of OVA (5 μg/mouse) containing 1 mg of adjuvant aluminum hydroxide (Pierce, Rockford, IL, USA) on days 0 and 7. Then, the mice were consecutively challenged three times intranasally with OVA (5 μg/mouse) and LPS (0.1 μg/mouse) ([2](#_ENREF_2)). For the inhibition experiments, mice received an oral dose of dexamethasone (1 mg/kg) or vehicle control (0.1 % methylcellulose) 1 h before every challenge.

***Semiquantitative reverse transcription PCR analysis***

Total RNA was extracted using easy-BLUE (iNtRON Biotechnology, Seoungnam, Korea). Then, 2 μg of extracted RNA was reverse-transcribed using Moloney murine leukemia virus reverse transcriptase (iNtRON Biotechnology), and BLT1, BLT2 and GAPDH were amplified using an RT-PCR PreMix kit (iNtRON Biotechnology). To analyze the transcripts, we identified the optimal PCR conditions for the linear amplification of GAPDH. The primers used were 5'-GCATGTCCCTGTCTCTGTTG-3' (forward), 5'-TCGGGCAAAGGCCTTAGTACG-3' (reverse) for BLT1; 5’-CAGCATGTACGCCAGCGTGC-3’ (forward), 5’-CGATGGCGCTCACCAGACG-3’ (reverse) for BLT2; 5'-CTGCACCACCAACTGCTTAGC-3' (forward), 5'-CTTCACCACCTTCTTGATGTC-3' (reverse) for GAPDH ([2](#_ENREF_2)). The specificity of all primers was confirmed by sequencing the PCR products.

1. ***Measurement of IL-17, LTB_4_ and 12(S)-HETE***
2. The amounts of IL-4, IL-5, IL-13, IL-17, LTB_4_ and 12(*S*)-HETE were quantified in BALF supernatant or serum using an ELISA kit (R&D Systems for IL-4, IL-5, IL-13 and IL-17; Enzo Life Sciences for LTB_4_ and 12(*S*)-HETE) according to the manufacturer’s instructions. BALF was obtained from mouse lungs using 0.65 ml of PBS after trachea cannulation, and the collected BALF was centrifuged at 1,000 × *g* for 3 min. Then, the supernatant was collected for ELISA.
3. ***Immunoblotting analysis***
4. Immunoblotting analysis was done as described previously ([3](#_ENREF_3)). Antibodies against 5-LO and 12-LO were obtained from BD Biosciences (Franklin Lakes, NJ, USA) and Santa Cruz Biotechnology (Santa Cruz, CA, USA), respectively. Antibodies against p-IκBα and β-actin (loading control) were obtained from Cell Signaling Technology (Danvers, MA, USA). Size estimates of proteins were obtained using molecular weight standards from Thermo Scientific (Rockford, IL, USA).
5. ***BAL cells and analysis of lung histology***
6. Inflammatory cells were collected from BALF by centrifugation (1,000 × *g* for 3 min) and washed with PBS. Next, BAL cells were fixed on glass slides and then stained with hematoxylin and eosin (H&E). Lung sections (5 μm thickness) were mounted onto Superfrost Plus glass slides (Fisher Scientific, Pittsburgh, PA, USA), deparaffinized and stained with H&E or periodic acid-schiff (PAS). A quantitative histological analysis to measure the degree of inflammation was performed by five independent, blinded investigators. The degree of peribronchial and perivascular lung inflammation was evaluated on a subjective scale from 0 to 3, as previously described ([4](#_ENREF_4)). Grade 0 indicated that no inflammation was detectable; grade 1 indicated the occurrence of occasional cuffing with inflammatory cells; grade 2 indicated that most bronchi or vessels were surrounded by a thin layer (one to five cells thick) of inflammatory cells; and grade 3 indicated that most bronchi or vessels were surrounded by a thick layer (more than five cells thick) of inflammatory cells. Images were acquired using a BX51 microscope (Olympus, Tokyo, Japan) equipped with a DP71 digital camera (Olympus).

***Calculation of percent reduction in inflammation***

Percent of reduction is calculated by finding the difference between value of OVA or LPS/OVA-control group and value of OVA or LPS/OVA-inhibitor group (value_control_-value_inhibitor_), and the result was divided by value of OVA or LPS/OVA-control group (value_control_). Lastly, the result was multiplied by 100. The formula was expressed as (value_control_-value_inhibitor_)/ value_control_ x 100.

1. ***Statistical analysis***
2. Student’s t-test was performed to assess the data for two groups, and Comparisons between more than three groups were performed with one‑way analysis of variance, followed by Tukey's post‑hoc test. SPSS software (IBM SPSS Statistics for Windows, version 21.0; IBM Corp., Armonk, NY, USA) was used for statistical analysis. In all experiments, 3 to 6 mice were included in each group. The results are presented as the mean ± SD. *P*-values <0.05 indicated statistical significance.
3. **Reference**
4. 1. Kim YK, Oh SY, Jeon SG, Park HW, Lee SY, Chun EY, et al. Airway exposure levels of lipopolysaccharide determine type 1 versus type 2 experimental asthma. J Immunol 2007;178(8):5375-5382.
5. 2. Lee AJ, Ro M, Cho KJ, Kim JH. Lipopolysaccharide/TLR4 stimulates IL-13 production through a MyD88-BLT2-linked cascade in mast cells, potentially contributing to the allergic response. J Immunol 2017;199(2):409-417.
6. 3. Ro M, Lee AJ, Kim JH. 5-/12-Lipoxygenase-linked cascade contributes to the IL-33-induced synthesis of IL-13 in mast cells, thus promoting asthma development. Allergy 2018;73(2):350-360.
7. 4. Cho KJ, Seo JM, Shin Y, Yoo MH, Park CS, Lee SH, et al. Blockade of airway inflammation and hyperresponsiveness by inhibition of BLT2, a low-affinity leukotriene B4 receptor. Am J Respir Cell Mol Biol 2010;42(3):294-303.

**Abbreviations**

BLT1, leukotriene B_4_ receptor-1; BLT2, leukotriene B_4_ receptor-2; LTB_4,_ leukotriene B_4_; 12(*S*)-HETE, 12(*S*)-hydroxyeicosatetraenoic acid; 5-LO, 5-lipoxygenase; 12-LO, 12-lipoxygenase; LPS, lipopolysaccharide; OVA, ovalbumin; BAL fluid, bronchoalveolar lavage fluid; Dexa, dexamethasone.
